# Improved Mechanical Properties of Ultra-High Shear Force Mixed Reduced Graphene Oxide/Hydroxyapatite Nanocomposite Produced Using Spark Plasma Sintering

**DOI:** 10.3390/nano11040986

**Published:** 2021-04-12

**Authors:** Bing-Yen Wang, Steven Hsu, Chia-Man Chou, Tair-I Wu, Vincent K. S. Hsiao

**Affiliations:** 1 Division of Thoracic Surgery, Department of Surgery, Changhua Christian Hospital, Changhua 500, Taiwan; 156283@cch.org.tw; 2 Center for General Education, Ming Dao University, Changhua 523, Taiwan; 3 School of Medicine, Chung Shan Medical University, Taichung 40201, Taiwan; 4 School of Medicine, College of Medicine, Kaohsiung Medical University, Kaohsiung 807, Taiwan; 5 Institute of Genomics and Bioinformatics, National Chung Hsing University, Taichung 402, Taiwan; 6 Ph.D. Program in Translational Medicine, National Chung Hsing University, Taichung 402, Taiwan; 7 School of Biomedical Engineering, Colorado State University, Fort Collins, CO 80523, USA; shsu13@rams.colostate.edu; 8 Division of Pediatric Surgery, Department of Surgery, Taichung Veterans General Hospital, Taichung 40705, Taiwan; 9 Department of Medicine, National Yang Ming Chiao Tung University, Taipei 112, Taiwan; 10 Department of Materials Engineering, Tatung University, Taipei 10451, Taiwan; tairiwu@yahoo.com.tw; 11 Department of Applied Materials and Optoelectronic Engineering, National Chi Nan University, Nantou 54561, Taiwan

**Keywords:** hydroxyapatite, graphene oxide, nanocomposite, ultra-high shear force, spark plasma sintering, mechanical property

## Abstract

The addition of nanomaterials, such as graphene and graphene oxide, can improve the mechanical properties of hydroxyapatite (HA) nanocomposites (NCPs). However, both the dispersive state of the starting materials and the sintering process play central roles in improving the mechanical properties of the final HA NCPs. Herein, we studied the mechanical properties of a reduced graphene oxide (r-GO)/HA NCP, for which an ultra-high shear force was used to achieve a nano-sized mixture through the dispersion of r-GO. A low-temperature, short-duration spark plasma sintering (SPS) process was used to realize high-density, non-decomposing r-GO/HA NCPs with an improved fracture toughness of 97.8% via the addition of 0.5 wt.% r-GO. Greater quantities of r-GO improve the hardness and the fracture strength. The improved mechanical properties of r-GO/HA NCPs suggest their future applicability in biomedical engineering, including use as sintered bodies in dentistry, plasma spray-coatings for metal surfaces, and materials for 3D printing in orthopedics.

## 1. Introduction

Recently, implant biomaterials have been studied closely for their tissue engineering applications [1]. Due to the complicated and variable environment of the human body, multi-functional implant biomaterials are necessary [2,3,4]. Traditional implant biomaterials, such as stainless steel, cobalt-based alloys, and metallic titanium [5], not only lack biocompatibility, but also often release metallic ions due to mechanical wear [6].

Hydroxyapatite (HA) is a biomaterial commonly used in surgical operations [7]. Due to its excellent biocompatibility, high biological activity and good osteoconduction, HA can be used in various applications, including in bone replacement and bone regeneration [8]. Likewise, HA exhibits lots of similarities with various inorganic minerals in the human body, leading to much research into its use as a potential replacement material for bone and teeth. In recent cases, HA has been used in tissue engineering for repairing or regenerating bone, and as a bioactive coating to improve the integration of implants into bone tissue [9,10]. Success has been achieved in using HA to promote the rapid growth of bone tissue in orthopedic surgery; however, long-term stability is still to be observed [11,12,13,14]. Similar to traditional ceramic materials, HA is brittle and less wear-resistant. The applicability of HA as an orthopedic biomaterial has also been limited due to its disparate mechanical properties when compared to natural bone [15]. To solve these issues, several studies have incorporated a secondary phase in order to improve some of the mechanical properties of HA [15,16,17,18,19].

The mechanical properties of ceramic materials can be improved by adding a particulate second phase [20,21]. The introduction of second phase materials into brittle ceramics has proven to be a promising method of improving toughness. This method can prevent the propagation of cracks in the ceramic material in various ways, leading to a drastic improvement in the flexural strength and fracture toughness of the composite material. Materials such as titanium alloy, alumina, and carbon nanotubes have commonly been used in the second phase to improve the electronic [22] or mechanical [14] properties of HA. The improvement of nanomaterials and nanotechnology has revolutionized ceramic toughening technology. Ceramics containing a nanostructure phase show a greatly increased number of grain boundaries due to grain refinement [20]. At the same time, the sizes of the pores and defects in such nanocomposites (NCPs) are reduced, leading to an increase in strength and toughness of the NCPs [21]. The mechanism for strengthening NCP ceramics works as follows: (1) The introduction of the dispersed phase effectively inhibits the growth of the grain matrix and reduces the abnormal growth of grains. (2) The dispersive second phase effectively stabilizes and strengthens the fine grain structure, thus promoting grain boundaries, which act as a barrier to crack propagation [21].

Although the mechanical properties of HA can be enhanced by the additional second phase, the inherent biological properties of HA and the activity of the adjacent tissue could be damaged by the addition of a second phase. In addition, some reinforcing materials, such as ZrO_2_, may cause HA to decompose during high-temperature manufacturing processes, resulting in a reduction in the biological activity [23]. Previous studies showed that carbon nanotubes (CNTs)/HA NCPs have greater fracture toughness compared to pure HA [24,25,26]; however, the biocompatibility of CNTs is still unclear due to their cytotoxic reactivity in organic environments. Although some researchers have claimed that the cytotoxic reactivity of CNTs is due to the metallic catalytic particles, and not the CNTs themselves [25], no consensus has been reached between various studies. It is still necessary to find an ideal secondary phase not only to enhance the mechanical properties of the host HA ceramic material, but also to maintain the biocompatibility of the HA in biological environments.

Since the discovery of graphene, its unique material properties in the context of electrical, thermal, optical, and mechanical applications have greatly impacted different research fields, such as electronics and biomedicine [27,28,29]. Some common methods of producing graphene include mechanical exfoliation, epitaxial growth, and chemical exfoliation. Recent studies have shown that graphene and graphene-based HA NCPs [30,31,32,33,34,35,36,37,38,39,40,41] have certain advantages, such as being non-toxic to human osteoblasts and having excellent antibacterial properties, as well as the ability to adhere to and proliferate osteoblasts, and to mineralize. The main purpose of this work is to use an ultra-high shear force [42] to mechanically peel off the reduced graphene oxide (r-GO). r-GO prepared via this method has an extremely low metal element content, making r-GO almost non-cytotoxic [43]. The use of an ultra-high shear force would allow for the simultaneous nano-scale mixing of r-GO and HA, resulting in uniform and stable r-GO mixing with the HA host material. Spark plasma sintering (SPS) at a low temperature and over a short time is then used to achieve the high-density, non-decomposing r-GO/HA NCPs with improved fracture toughness. We conclude that the realization of an optimal addition ratio of r-GO to HA, and the mechanical properties that are thus achieved, could contribute to the development of effective graphene/HA NCPs for biomedical applications.

## 2. Materials and Methods 

### 2.1. Materials and Dispersion Process

The r-GO (Cheap Tubes Inc., Cambridgeport, VT, USA) of 300–800 nm with a horizontal thickness of 0.7–1.2 nm was fabricated via the improved Hummer method. By reducing the graphene oxide using a microwave process, we can remove the functional groups and restore the carbon structure. The HA powders with a particle size of 60 nm were obtained from Yu-Ching Co., Hsinchu, Taiwan. The r-GO/HA mixture was achieved by mixing 3 g r-GO in powder form and 200 g HA in powder form into 100 mL and 500 mL of N-Methylpyrrolidone (NMP) solvent, respectively. The mixture was then treated in an ultra-high-pressure homogenizer for 5 cycles of the dispersion treatment. The ultra-high shear force required for mixing r-GO and HA was generated using the ultra-high-pressure homogenizer (NVL-ED) from Yoshidakikai Co., Nagoya, Japan. Via normal dispersion processing, the performance quality of the dispersed particles may be reduced due to the damaged particle surface. Here, we used wet processing, wherein the slurry was directed to the nozzle through a high-pressure plunger pump. The maximum flow rate was 290 mL/s when the maximum processing pressure was 200 MPa. Homogeneous dispersion can be achieved by controlling the power, which enables very fine distribution. The nozzle used here was a cross (X-type) nozzle. This nozzle design requires large amounts of processing energy and is mainly used for crushing and dispersion processing. The material of the nozzle was single crystalline diamond so as to avoid contamination and severe wear.

### 2.2. Characteristics of Ultra-High Shear Force-Treated r-GO/HA NCP 

The X-ray diffraction (XRD) patterns of the NCP were obtained in a 2*θ* range of 0–90° on a high-resolution X-ray diffractometer. Transmission electron microscopy (TEM) was performed using a JEOL JEM-2100 TEM (JEOL, Tokyo, Japan). Raman measurements were taken using a micro-Raman system (LabRAM HR800, HORIBA Taiwan Inc., Hsinchu, Taiwan) with a helium–neon laser as the excitation source, operating at a 633 nm wavelength and equipped with a 40× objective lens. Scanning electron microscopy (SEM) was conducted using a JEOL JSM-7800 F field-emission SEM (JEOL, Tokyo, Japan). The thickness of the r-GO was measured using a non-contact Fizeau laser interferometer (ZYGO Verifire™ ATZ, AMETEK Taiwan Corp., LTD, Hsinchu, Taiwan).

### 2.3. Spark Plasma Sintering Process 

Spark plasma sintering (SPS) [25] is a solid compression-sintering method similar to hot press sintering. However, the method of heating that SPS involves is different from that in hot press sintering. Plasma discharge sintering involves directly applying an on–off dc pulsed voltage and current to the sintered mold. The self-heating of the sintering mold to which electric energy is directly applied (or the material itself, if it is a conductive material) is the driving force of the sintering, together with pressure. As the heating range is limited, the heating and cooling speeds are faster (several minutes to hours) compared with hot press sintering. Via pressurization and rapid temperature increases, a dense sinter with a suppressed growth of crystal grain can be obtained. Sintering is carried out under vacuum conditions of several pascal, and the outer peripheral temperature of the sintering mold is measured via the observation window of the vacuum chamber with an infrared thermometer. The SPS process was carried out at and room temperature with a magnet stirrer for 24 h. The SPS equipment was the LABOX-325R model from SinterLand Inc. (Nagaoka, Japan).

### 2.4. Mechanical Characterization

Bending strength analyses were performed with a universal testing machine (UTM, Alpha Precision Instrumentation Corp., Taipei, Taiwan). The test samples of 10 bars for each NCP were prepared by filling a graphite mold with a diameter of 50.8 mm with r-GO/HA. After a circular cake-shaped sample was formed via SPS, the surface was trimmed with a surface grinder, and the sample was then cut with a very slow cutting machine. The tested samples had a length of 35 mm, a width of 4 mm, and a height of 4 mm. The sintered body was tested with a UTM to measure the compressive and bending strengths using a pressing fixture and a three-point bending fixture, at 1.5 mm/min and with a maximum down force of 300 kg.

## 3. Results and Discussion

### 3.1. Surface Morphology of Dispersive r-GO/HA Nanocomposite

**Figure 1:**
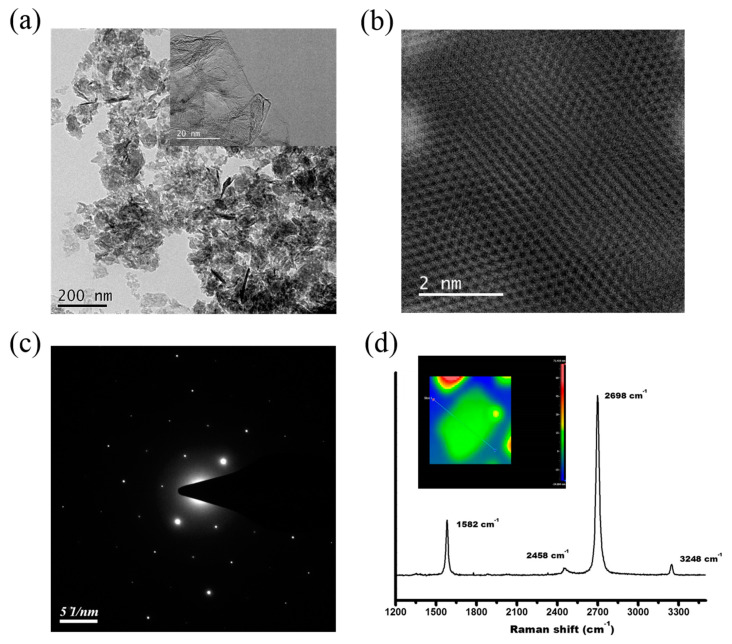
(**a**) Transmission electron microscopy (TEM) micrograph of ultra-high shear force-treated reduced graphene oxide hydroxyapatite nanocomposites (r-GO/HA NCPs). The inset shows the r-GO with only a few layers. (**b**) High-angle annular dark-field (HAADF) image of r-GO. (**c**) Electron diffraction pattern of r-GO. (**d**) Raman spectrum of ultra-high shear force-treated r-GO. The inset shows the layer thickness of the r-GO to be around 15 nm.

Figure 1 shows the physical characteristic of the r-GO after treatment with an ultra-high shear force. The TEM image of the mixture shows that the slightly wrinkled r-GOs were well-dispersed in the HA. The inset of Figure 1a shows that the r-GO was well layered with a larger sheet diameter structure. Figure 1b shows the atomic arrangement of the r-GO, with a typical hexagonal structure of carbon atoms. The high-angle annular dark-field (HAADF) image of the r-GO (Figure 1c) suggests that the diffraction pattern of the r-GO is hexagonal, implying its graphite structure, and the bright spots are not deformed, indicating that the graphene structure has not been damaged after the ultra-high-pressure homogenization treatment. Figure 1d shows the Raman spectrum of r-GO after ultra-high-pressure homogenization treatment. The G and 2D bands’ peaks are strong, and the D band is lower than the G band, indicating that the structure of the graphite is undamaged. The peak position of the 2D band is below 2700 cm^−1^, indicating that the number of graphite layers is less than five [43]. Previous studies [44] have proven that the intensity ratio of the 2D and G bands should be above 1.3 for chemical vapor-deposited single-layer graphene. Here, for the r-GO treated with ultra-high-pressure homogenization, the intensity ratio of the 2D and G bands is 1.37. Using a non-contact Fizeau laser interferometer, the thickness of the graphene after ultra-high-pressure homogenization treatment was measured, as shown in Figure 1d (inset). Since the thickness of the single-layered r-GO is between 1.3 and 3.0 ± 0.2 nm, the ultra-high shear force-treated r-GO is estimated to have 5–7 layers, which is similar to the result of the Raman measurement.

**Figure 2:**
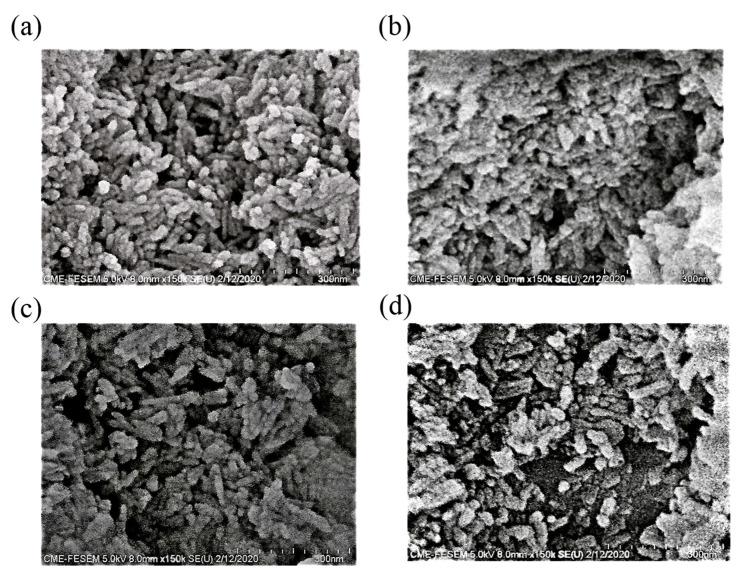
Scanning electron microscopy (SEM) micrograph of un-sintered (**a**) HA, and the (**b**) 0.1 wt.% r-GO/HA, (**c**) 1 wt.% r-GO/HA, (**d**) and 5 wt.% r-GO/HA nanopowders.

The r-GO homogenized at an ultra-high pressure was added into the HA solution, which was again mixed via ultra-high-pressure homogenization with various adding ratios of r-GO concentration (0.1 wt.%, 0.5 wt.%, 1 wt.%, 2 wt.% and 5 wt.%). Five different concentrations of r-GO solution (0.1 wt.%, 0.5 wt.%, 1 wt.%, 2 wt.% and 5 wt.%) were assessed for their effects on mechanical properties. The r-GO solutions, all of which were homogenized at ultra-high pressures, were individually mixed with an HA solution. The newly formed r-GO/HA mixture was then homogenized again at an ultra-high pressure to ensure uniform mixing. After the mixing process, the slurry was vacuum-distilled to remove the solvent, and the dried r-GO/HA NCPs were ground and sieved (screen mesh: 100 mesh). The powder after sieving is shown in Figure 2; the particle size of the powder after grinding and sieving was about 10–40 microns, depending on the additional concentration of r-GO.

### 3.2. Pore Size and Density of SPS Treated r-GO/HA Nanocomposite

The SPS temperature was set at 1000 °C, and the pores were encapsulated via vacuum evacuation (less than 10 Pa). At this point, the upper and lower electrodes applied a pre-pressure of 25 MPa to the sinter in the mold, and then started electric heating. The SPS temperature was increased to 600 °C, then held for 5 min. This was to ensure that any organic impurities from previous processes were completely evaporated, as well as to avoid forming closed pores during the pre-pressing process. After the temperature was held at 600 °C for 5 min, the pressure of the upper and lower electrodes was increased to 50 MPa and the temperature was increased to 1000 °C at a heating rate of 100 °C/min. After holding the temperature at 1000 °C for 10 min, the samples were cooled in a vacuum to below 150 °C. The sinters were then removed for subsequent analysis and mechanical performance testing.

The two-inch diameter sinters were further ground to obtain a smooth surface that could facilitate the measurements of true density and porosity as the r-GO is added. The density and porosity of the sintered samples were measured via the buoyancy method following the Archimedean principle, the boiling water method, the wax-sealing method and the vacuum saturation method. The measured results are shown in Table 1. As the amount of added r-GO increases, the porosity also increases. This is because r-GO inhibits the grain growth of HA crystals, resulting in pores arising between grains. There is no way to eliminate the pores between grains via atomic diffusion or grain growth mechanisms. In addition, the porosities are all controlled to below 1% with the addition of r-GO, suggesting that SPS is suitable for preparing high-density r-GO/HA NCPs at lower sintering temperatures and through shorter sintering periods. 

### 3.3. Characterization of SPS-Treated r-GO/HA Nanocomposite

**Figure 3:**
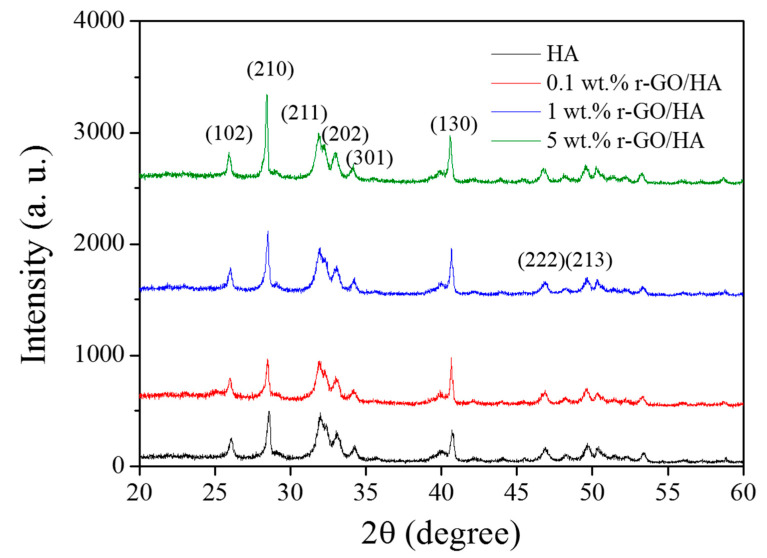
X-ray diffraction (XRD) of sintered HA and r-GO/HA NCPs with different concentrations of r-GO.

X-ray diffraction (XRD) analyses were carried out on the sintered r-GO/HA NCPs (Figure 3). The result indicates that the composition of the sintered NCPs is very close to that of the original un-sintered HA. The lack of an obvious carbon peak in the r-GO indicates that no phase transformation of r-GO occurred during the SPS process, which means that the HA itself neither underwent chemical reactions during SPS nor reacted with r-GO to form carbides.

### 3.4. Mechanical Properties of SPS-Treated r-GO/HA Nanocomposite

After measuring the densities and porosities of the sinters, they were molded into an elongated shape with the following dimension: 35 mm length, 4 mm width and 4 mm height. The surface of the sinters was then abraded with sandpaper in running water. Lastly, the surface was polished using diamond paste to minimize the possible defects that could significantly alter the measurement data. The three-point flexural failure strengths of the sintered specimens were tested under the conditions of 1.5 mm/min and a maximum down force of 150 kg, using a UTM with a down-compression fixture and a three-point anti-folding fixture. The results are shown in Table 2. When the sample contains 1 wt.% of r-GO, the bending strength of the r-GO/HA NCP can reach 402.56 MPa, which is twice that of the original HA sample. When the sample contains 5 wt.% of r-GO, the bending strength of the r-GO/HA NCP is 215.19 MPa, which is only 9.8% higher than that of the original HA sample. A possible explanation is that excess r-GO gathering near the grain boundary, causing irreversible damage. Additionally, the addition of excess r-GO may increase the number of pores, leading to reduced fracture toughness. Since the starting material is in powder form, residual pores would remain in the grain boundaries after the sintering process. This, in turn, affected the mechanical properties of the r-GO/HA NCPs sinters.

A load test force of 0.5 Newtons was employed to measure the Vickers hardness of the sintered specimens. A previous study showed that the Vickers hardness increases with an increasing load [45]. At low loading rates, the energy is spent on plastic and elastic deformation. However, at high loading rates, the energy is spent on elastic deformation, plastic deformation, nucleation, and the growth of cracks. The results, reported in Table 2, show that the addition of r-GO could help in improving the mechanical properties of HA. For example, when the sample contained 1 wt.% r-GO, the Vickers hardness reached 525.12, which is 65.6% higher than the pure HA sample. When the concentration of r-GO was increased to 5 wt.%, the Vickers hardness was 457.42, which is 44.1% higher than that of the pure HA sample. This indicates that that Vickers hardness is inversely proportional to the grain size. However, as the amount of r-GO added exceeds 1 wt.%, the pores may not be easily eliminated, resulting in a reduction in Vickers hardness.

Through the result of the measurement of Vickers hardness, the fracture toughness, K_IC_, can be calculated using the median crack system [46],

(1) $$\begin{array}{c} K_{\mathrm{IC}}=\frac{0.0726P}{c^{1.5}} \end{array}$$

where P is the applied load (N), and c is the length of the crack from the center of the indentation to the tip of the crack (mm). The results show that when 0.5 wt.% of r-GO was added to HA, the fracture toughness could reach 6.21 MPa m^1/2^, which is 97.8% higher than that of pure HA. When the amount of r-GO was increased to 5 wt.%, the fracture toughness reached 3.89 MPa m^1/2^, which is 24.2% higher than that of pure HA. Increasing the amount of r-GO first helps increase the fracture toughness; however, when the addition of r-GO exceeds 0.5 wt.%, a crack appears along the grain boundary and a so-called epitaxial failure forms, and intragranular failures also arise that split the crystal grains. Furthermore, the addition of r-GO graphene may have a bridging effect on the cracks.

### 3.5. Analysis of Surface Morphology of SPS-Treated r-GO/HA Nanocomposite

**Figure 4:**
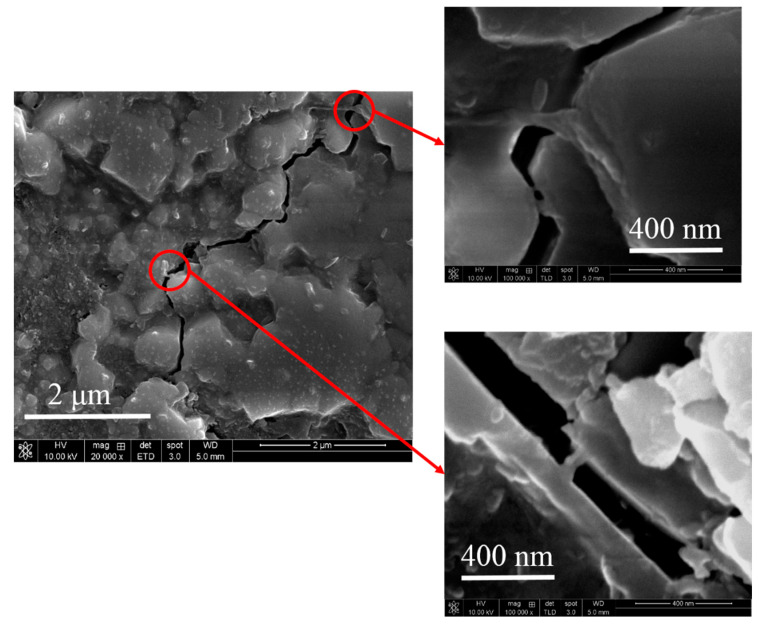
SEM micrograph of sintered 1 wt.% r-GO/HA showing the presence of r-GO for bridging the cracks.

From the mechanical measurement data, we can infer that the three mechanical properties reach their highest values when the amount of graphene added is 0.5–1 wt.%; however, the values of the mechanical properties begin to deteriorate as the amount of r-GO added exceeds 1 wt.%. When the amount of added r-GO is less than 1wt.%, the r-GO between the HA particles inhibits the growth of HA crystal grains, such that the sintered body exhibits grain refinement and the cracks progress. The process gives rise to twists and turns, as shown in Figure 4, which absorb the energy that causes damage, thus strengthening the compound. The r-GO between the grain boundaries exerts a mechanical force between the HA grain boundaries. Since the r-GO has optimal mechanical properties, the addition of r-GO halts the propagation of cracks and absorbs the energy of partial destruction, thus improving the mechanical properties of the r-GO/HA NCPs. The bridging effect of r-GO within the HA matrix is shown in Figure 4 (magnified images). However, as the amount of r-GO exceeds 1 wt.%, the amount of r-GO between the HA grain boundaries increases. Since the r-GO could not chemically bond with HA, the increased content of r-GO inside the grain boundary weakens the mechanical force, leading to a reduction in the mechanical properties of r-GO/HA NCPs. In addition, as the amount of added r-GO increases, the number of internal closed pores also increases, further weakening the mechanical properties of the material.

**Figure 5:**
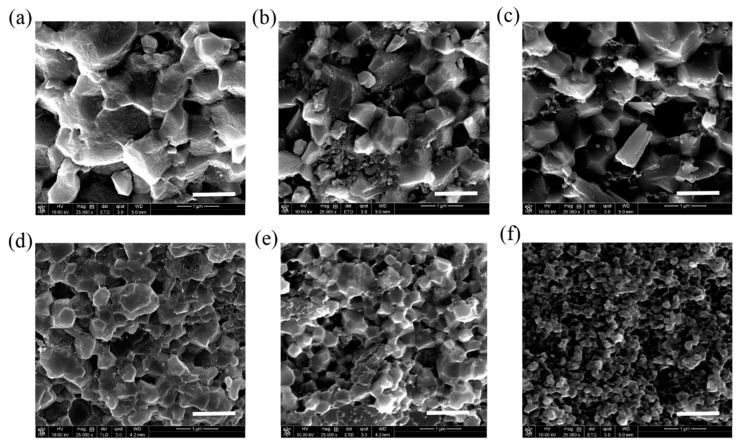
SEM micrograph of sintered (**a**) HA, (**b**) 0.1 wt.% r-GO/HA, (**c**) 0.5 wt.% r-GO/HA, (**d**) 1 wt.% r-GO/HA, (**e**) 2 wt.% r-GO/HA, and (**f**) 5 wt.% r-GO/HA NCPs. The scale bar is 1 μm.

**Figure 6:**
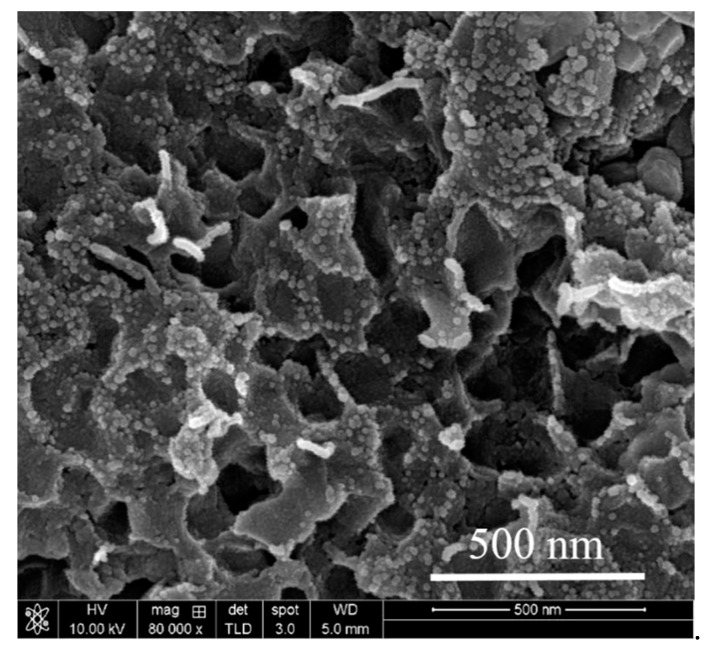
SEM micrograph of sintered 5 wt.% r-GO/HA NCPs with higher magnification.

Figure 5 shows the SEM images of the specimen’s cross-section after the failure of the three-point flexural strength tests. As the amount of added r-GO increases, the grain size shrinks. In addition, when the quantities of added r-GO are 0 wt.%, 0.1 wt.% and 0.5 wt.%, abnormal grain growth occurs. The reason for this is that, during the plasma sintering process, a significant current passes through the sample via the cavity with lowest resistance. A higher temperature is generated in this area because the current is greater during the sintering process, causing the HA to melt and accelerating the growth of crystal grains. When the amount of r-GO added exceeds 1 wt.%, the HA crystal grains become smaller and more uniform, and holes appear. This is because the grain growth of HA is inhibited by the added r-GO. The higher the amount of added graphene, the more obvious the effect of inhibition becomes. As Figure 6 shows, a large amount of r-GO gathers on the HA grain boundary. Since the r-GO does not form a chemical bond with HA, this r-GO accumulates on the grain boundary, which is similar behavior to what would be seen with a large number of nanopores. The buildup of r-GO on the grain boundary leads to localized instability, resulting in a reduction in mechanical strength.

## 4. Conclusions

We have successfully fabricated high-density, non-decomposing r-GO/HA NCPs with improved fracture toughness. Ultra-high shear force was used to realize the nano-sized r-GO that was dispersed in the HA matrix. The low-temperature, short-duration spark plasma sintering process was used to achieve r-GO/HA NCPs with fracture toughness of 97.8% via the addition of 0.5 wt.% r-GO. The improved mechanical properties of the r-GO/HA NCPs suggest this process’ applicability in biomedical engineering, such as its use for sintering bodies in dentistry, plasma spray-coating on metal surfaces, and 3D printing in orthopedics.

## Figures and Tables

**Table 1:** Geometrical and physical properties of HA and r-GO/HA NCPs.

r-GO (wt.%)	0.1	0.5	1.0	2.0	5.0
Diameter (mm)	50.8 ± 0.2	50.8 ± 0.2	50.8 ± 0.2	50.8 ± 0.2	50.8 ± 0.2
Height (mm)	5.33 ± 0.05	5.37 ± 0.05	5.36 ± 0.05	5.38 ± 0.05	5.40 ± 0.05
Weight, dry (g)	32.82 ± 0.05	32.99 ± 0.05	32.83 ± 0.05	32.82 ± 0.05	32.82 ± 0.05
Weight, wet (g)	32.75 ± 0.05	32.76 ± 0.05	32.79 ± 0.05	32.80 ± 0.05	32.80 ± 0.05
Weight, water (g)	22.07 ± 0.05	22.01 ± 0.05	22.07 ± 0.05	22.06 ± 0.05	22.04 ± 0.05
Apparent density (g/cm^3^)	3.040 ± 0.020	3.033 ± 0.020	3.023 ± 0.020	3.011 ± 0.020	3.000 ± 0.020
True density (g/cm^3^)	3.073 ± 0.020	3.069 ± 0.020	3.061 ± 0.020	3.057 ± 0.020	3.050 ± 0.020
Porosity (%)	0.226 ± 0.050	0.362 ± 0.050	0.605 ± 0.050	0.737 ± 0.050	0.968 ± 0.050

**Table 2:** The mechanical properties of HA and r-GO/HA NCPs with different concentrations of r-GO.

Sample	Three Point Bending Strength (MPa)	Vickers Hardness (Mpa)	Fracture Toughness, K_IC_ (MPa m^1/2^)
HA	195.48 ± 12.70	317.45 ± 3.22	3.14 ± 0.52
0.1 wt.% r-GO/HA	225.67 ± 14.21	355.58 ± 3.70	4.16 ± 0.34
0.5 wt.% r-GO/HA	315.38 ± 16.22	483.60 ± 5.13	6.21 ± 0.17
1.0 wt.% r-GO/HA	402.56 ± 19.14	525.12 ± 5.75	5.80 ± 0.30
2.0 wt.% r-GO/HA	303.75 ± 15.38	506.57 ± 5.25	4.42 ± 0.42
5.0 wt.% r-GO/HA	215.19 ± 12.12	457.42 ± 4.83	3.89 ± 0.45

## Data Availability

The data presented in this study are openly available in https://www.mdpi.com.
